# Bibliometric analysis of global research trends and prospects on circulating tumor DNA in colorectal cancer

**DOI:** 10.3389/fonc.2024.1428942

**Published:** 2024-11-15

**Authors:** Jing Pang, Pengyu Bai, Yong Zhang, Lichun Wang

**Affiliations:** Shanxi Province Cancer Hospital/Shanxi Hospital Affiliated to Cancer Hospital, Chinese Academy of Medical Sciences/Cancer Hospital Affiliated to Shanxi Medical University, Taiyuan, China

**Keywords:** circulating tumor DNA, colorectal cancer, CtDNA, bibliometric analysis, CRC

## Abstract

**Background:**

Colorectal cancer (CRC) is one of the most commonly diagnosed advanced-stage malignancies worldwide and places a substantial burden on both the economic and social development of numerous countries.

**Objective:**

This manuscript aims to synthesize the existing evidence and explore potential avenues for future scholarly research on ctDNA in CRC.

**Methods:**

Bibliometric analyses were performed using the bibliometrix package in R, along with CiteSpace and VOSviewer software. The search was restricted to publications up to 31 March 2024, using the following terms: (“ctDNA” OR “circulating tumor DNA”) AND (“colorectal cancer” OR “colorectal tumor”) from the Web of Science Core Collection (WoSCC) database.

**Results:**

Ultimately, we identified 1,310 documents published in 353 journals authored by 7,683 researchers from 2,417 institutions across 66 countries. The USA was the most productive country. The *Journal of Clinical Oncology* was the most prolific, publishing 111 articles with 3,396 citations. The top five keywords were “colorectal cancer,” “circulating tumor DNA,” “acquired resistance,” “cell-free DNA,” and “plasma.” The top five cluster labels for references were “advanced cancer,” “metastatic colorectal cancer,” “liquid biopsy,” “colorectal cancer,” and “human colorectal cancer xenograft.”

**Conclusions:**

The collaborative networks are primarily composed of highly productive authors, prestigious institutions, and leading countries. Additionally, the advancement of detection technologies, the development of standardized protocols, the exploration of circulating tumor DNA (ctDNA) dynamics in CRC, and the implementation of large-scale clinical trials for ctDNA-guided precision therapy in CRC are expected to become major research priorities in the future.

## 1 Introduction

The incidence of colorectal cancer (CRC), a common advanced-stage malignancy worldwide, is rising annually in developing countries due to comparatively low screening coverage and participation rates. According to the International Agency for Research on Cancer (IARC), approximately 1,935,500 new cases of CRC were reported in 2022. CRC ranks as the second leading cause of cancer-related deaths, accounting for an estimated 895,000 fatalities (1). Circulating tumor DNA (ctDNA), which contains distinct genetic and epigenetic markers of the tumor, is released into the bloodstream through tumor cell apoptosis or necrosis. In recent decades, a growing number of researchers have focused on using ctDNA for early detection of CRC, developing adjuvant therapies for stage II/III CRC, and assessing treatment efficacy in metastatic CRC.

Bibliometric analysis involves quantitatively evaluating scientific publications and their citations by constructing a graph representing the network of inter-document citation relationships. While this method has been widely used to analyze the status and evolution of publications in the medical sciences, to the best of our knowledge, no formal citation analysis has been conducted on ctDNA in the context of CRC (2, 3). Understanding the existing body of evidence and predicting future research directions are crucial. The use of visualization techniques and bibliometric analysis aids in identifying developmental trends in research related to CRC-associated ctDNA.

## 2 Methods

### 2.1 Data source and search strategy

We conducted a search in the Web of Science Core Collection (WoSCC) database, with the publication date restricted to 31 March 2024. The following search terms were used: (“ctDNA” OR “circulating tumor DNA”) AND (“colorectal cancer” OR “colorectal tumor”). The inclusion criteria encompassed published records. The data categories included articles, review articles, meeting abstracts, editorial material, early access, and proceedings papers. Corrections, book chapters, letters, news items, publications with expressions of concern, retractions, retracted publications, and manuscripts in languages other than English were excluded. Two reviewers worked independently, and a third reviewer resolved any disagreements. The data were saved in the format “Plain Text with Full Record and Cited References” and imported into the analysis software.

### 2.2 Productivity analysis

The “bibliometrix” package in R was employed to analyze fundamental publication characteristics, including author information, author affiliations, countries of origin, journal sources, keywords, and other related metrics.

### 2.3 Comprehensive analysis

The software VOSviewer (version 1.6.18) was used to visualize the relationships between countries, institutions, authors, keywords, and references by generating social network maps (4). We also utilized CiteSpace (version 6.3.R1 Advanced) to analyze the social network of knowledge foundations, research hotspots, development trends, and key literature on this topic. Cluster analysis, which reveals the structure of research fields and identifies research hotspots and trends, along with co-citation analysis, where both A and B are cited by C, was conducted using VOSviewer and CiteSpace. Burst detection was employed to identify hot keywords or references that showed a sudden increase in frequency during a particular period. Cluster dependency was used to express the direction and mode of knowledge flow. Both analyses were carried out with CiteSpace (5). Social network maps consist of nodes and lines. Nodes of varying colors and sizes represented differences in the frequency of document characteristics in different clusters, while the links between nodes indicated the intensity of collaboration (6).

## 3 Results

### 3.1 General information

**Figure 1:**
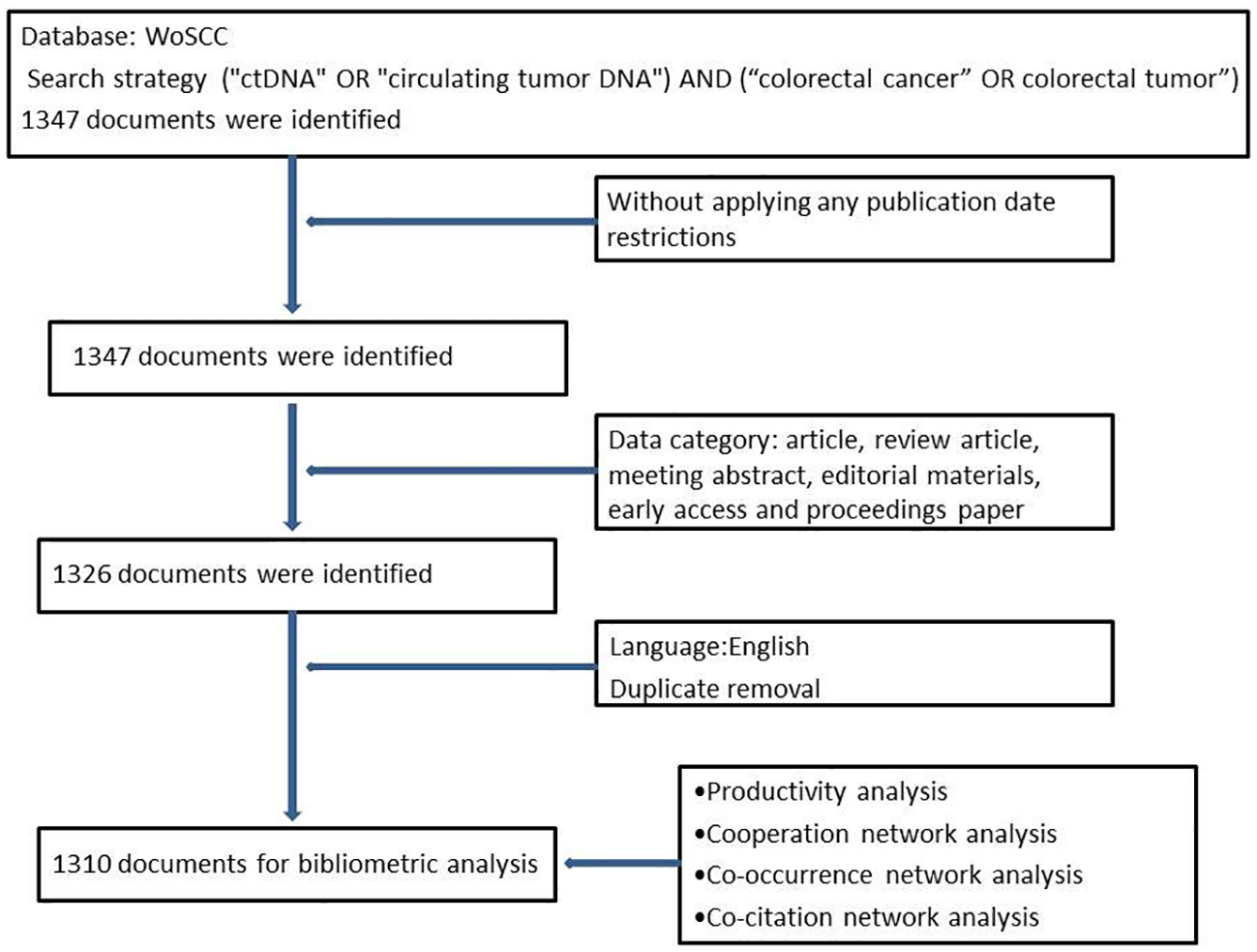
The process of literature search and screening.

A total of 1,310 documents were retrieved from the WoSCC for bibliometric analysis. The literature search and screening process is illustrated in **Figure 1**. All 1,310 documents were published across 353 sources. The average age of the documents was 3.94 years, with an average of 35.49 citations per document. In total, 30,008 references and 7,683 authors were identified. The number of author keywords and Keywords Plus was 1,460 and 1,536, respectively. On average, there were 10.5 authors per document. The percentage of international co-authorships was 27.1%. The collection included 661 articles and 348 reviews, representing 50.46% and 26.57% of the total, respectively (**Table 1**).

**Table 1:** Detailed information on the data included.

Description	Results
Timespan	2000–2024
Sources (journals, books, etc.)	353
Documents	1,310
Annual growth rate %	15.68
Average age of the document	3.94
Average citations per doc	35.49
References	30,008
Document Content	
Keywords plus (ID)	1,536
Author keywords (DE)	1,460
Authors	
Authors	7,683
Authors of single-authored docs	27
Author Collaboration	
Single-authored docs	27
Co-authors per doc	10.5
International co-authorships %	27.1
Document types	
Original article	661
Editorial materials	27
Editorial materials; early access	1
Conference abstract	272
Proceedings paper	1
Review	348

### 3.2 Productivity analysis

**Figure 2:**
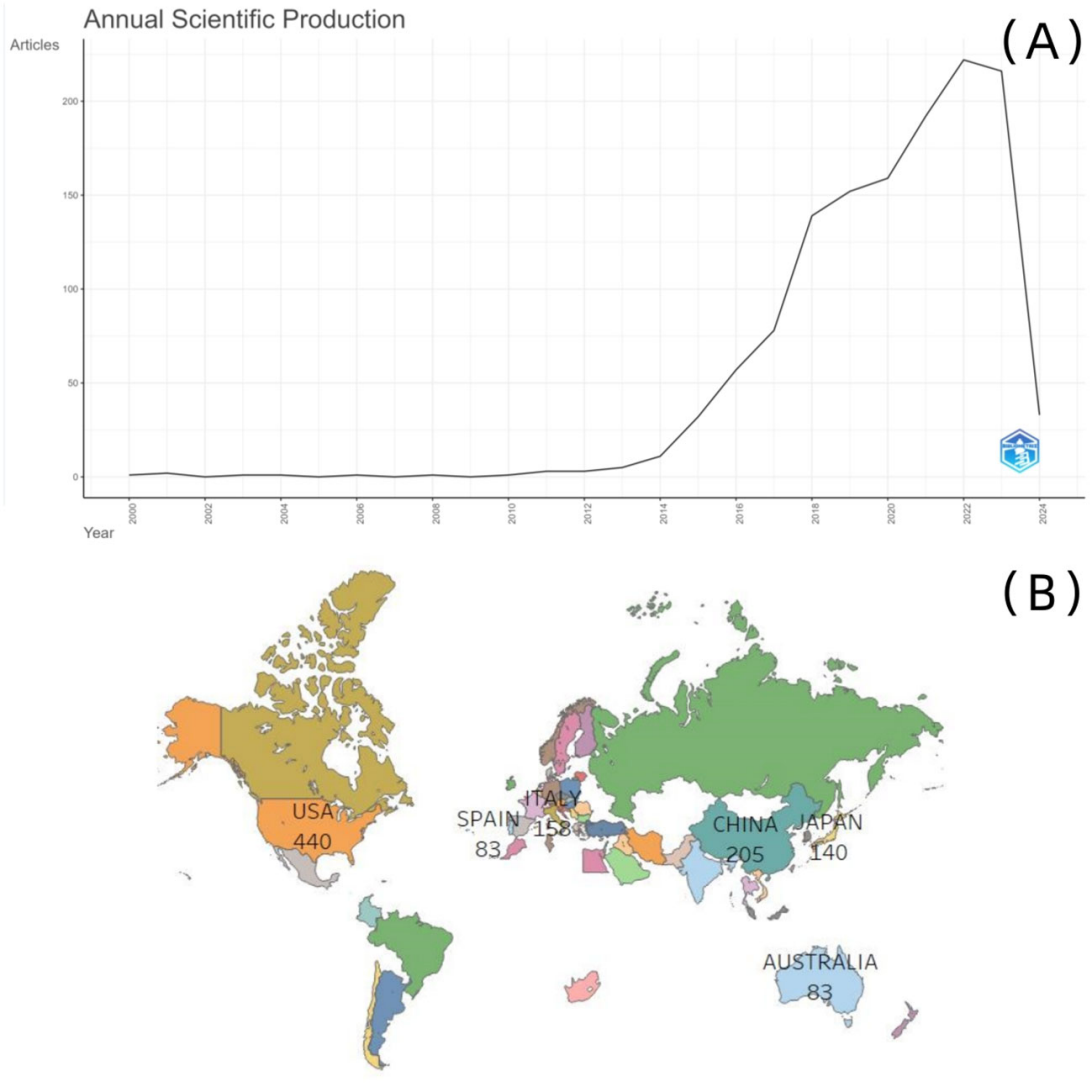
**(A)** Annual scientific production; **(B)** scientific production by country.

The number of published documents from 2000 to 2013 remained stable, followed by a sharp increase over the last decade. The highest number of articles published was 222 in 2022 (**Figure 2A**). Despite the COVID-19 pandemic from 2020 to 2022, researchers’ enthusiasm for ctDNA associated with CRC was not diminished. More than half of the documents (663 out of 1,310) were published between 2021 and 1 March 2024 (**Supplementary Table 1**).

Scholars from 66 countries worldwide have demonstrated an interest in ctDNA associated with CRC. The USA had the highest number of publications, making it the most productive country overall. However, when accounting for the number of researchers and normalizing publication output, Italy emerged as the country with the most significant contribution (**Figure 2B**; **Table 2A**). The most productive journal in this field was the *Journal of Clinical Oncology*, which also held the highest citation count (111 articles, 3,396 citations). Furthermore, it was identified as a core publication based on Bradford’s law. The top five most productive journals, along with their citation counts and H-index (a metric used to evaluate both the quantity and impact of academic output), are shown in **Table 2B**.

**Table 2A:** The five most productive countries.

Rank	Country	Count*/authors	Articles**	Citations	Average article citations
1	USA	0.1399 (440/3,145)	282	12,265	43.50
2	China	0.1368 (205/1,498)	206	3,147	15.30
3	Italy	0.1196 (158/1321)	107	6,769	63.30
4	Japan	0.1832 (140/764)	84	1,831	21.80
5	Spain	0.1023 (83/811)	41	1,992	48.60
5	Australia	0.1350 (83/615)	51	3,619	71.00

*Count: Full count, in academic collaborations involving the authorship of literature, each co-author is accorded a score that is consistent and equivalent to the total value attributed to that literature.

**Articles: The volume of national publications was ascertained by enumerating the countries associated with corresponding authors.

**Table 2B:** Top five most productive journals.

Rank	Journal	Articles	Citations	H-index
1	*Journal of Clinical Oncology*	111	3,396	21
2	*Annals of Oncology*	81	2,967	20
3	*Cancers*	77	654	16
4	*Cancer Research*	52	1,154	2
5	*Frontiers in Oncology*	41	384	8

### 3.3 Comprehensive analysis

#### 3.3.1 Cooperation network analysis

**Figure 3:**
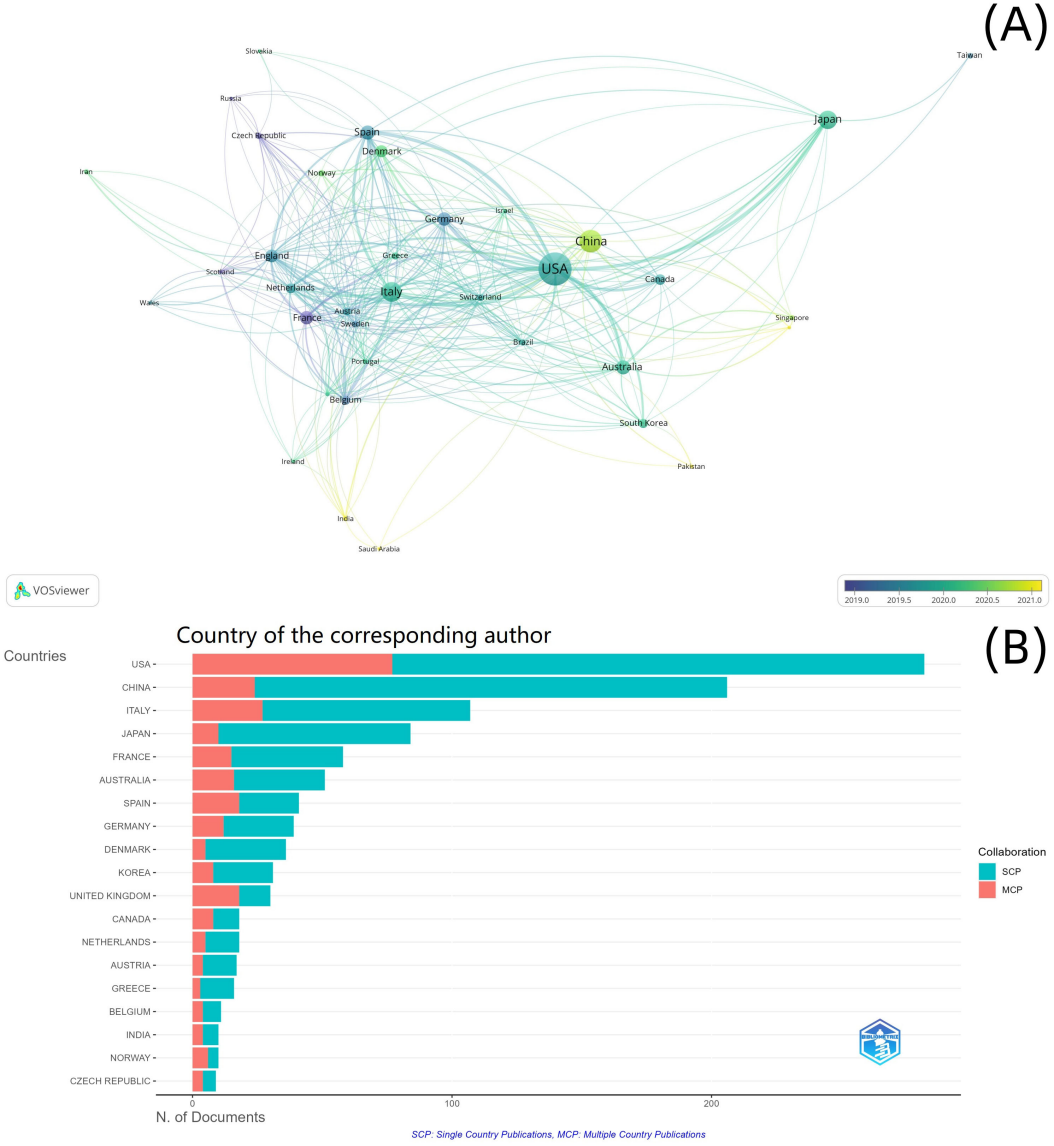
National cooperation analysis. **(A)** National cooperation network; **(B)** country of the corresponding author.

In the map and overlay visualization of the national collaborative networks, based on the top 36 frequencies, seven clusters were formed (**Figure 3A**). The most productive countries in each cluster were France (*n* = 77, links = 25, total link strength = 87, Cluster 1), USA (*n* = 443, links = 34, total link strength = 380, Cluster 2), Spain (*n* = 84, links = 25, total link strength = 133, Cluster 3), Italy (*n* = 158, links = 29, total link strength = 178, Cluster 4), Australia (*n* = 84, links = 23, total link strength = 90, Cluster 5), Greece (*n* = 20, links = 21, total link strength = 36, Cluster 6), and Japan (*n* = 140, links = 20, total link strength = 72, Cluster 7). The largest cluster was Cluster 1, which consisted of 10 nodes representing different countries (**Figure 3A**). The USA had 77 multiple-country publications, accounting for 21.69% of the total number of multiple-country publications (**Figure 3B**).

**Figure 4:**
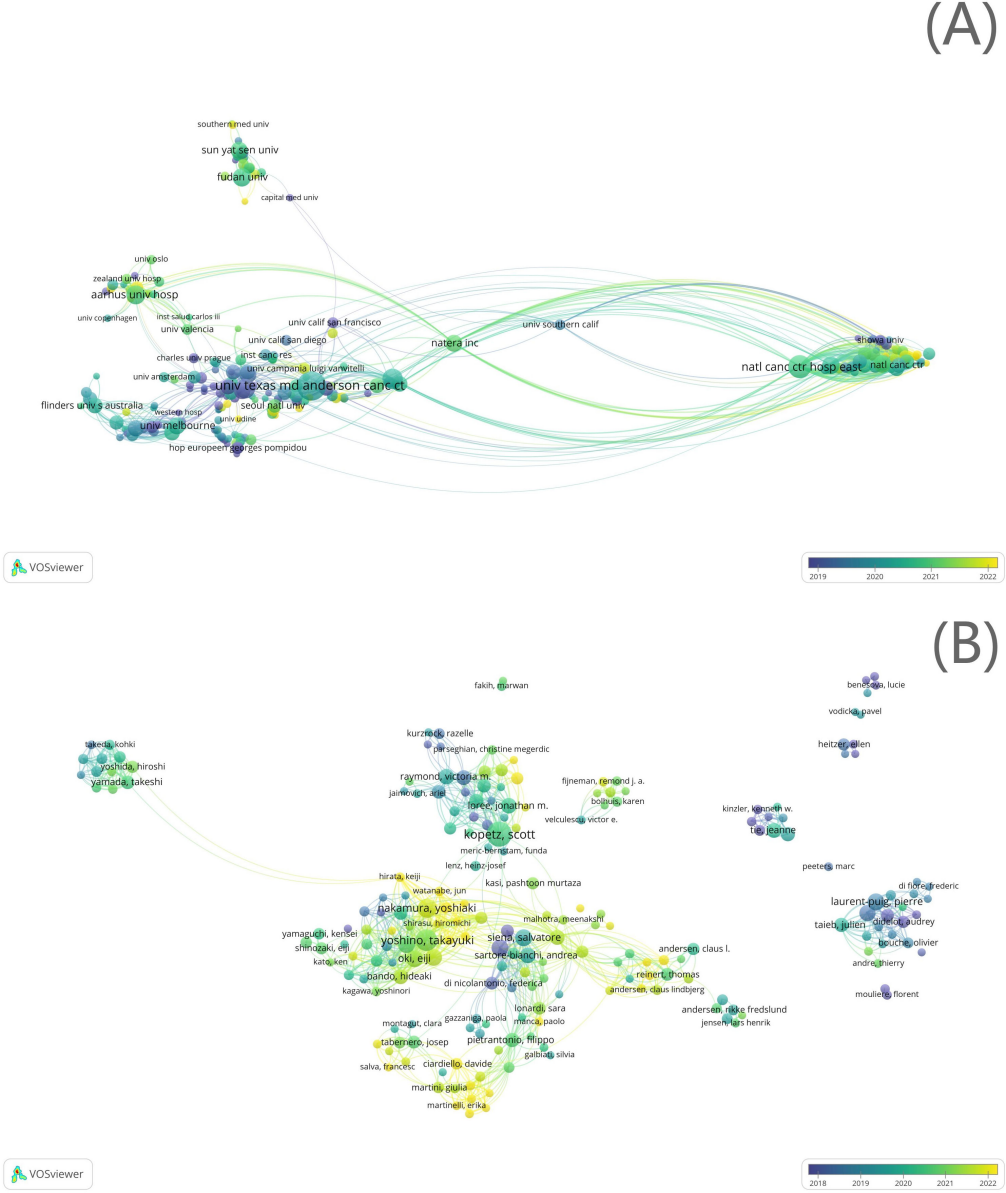
Institutional cooperation analysis. **(A)** Institutional cooperation network; **(B)** author cooperation network.

Among the 2,417 institutions, 220 institutions with more than five documents were included in the cooperation network analysis. Eight clusters were formed in the institutional cooperation network map and overlay visualization (**Figure 4A**). The most productive institutions in the top three clusters were National Cancer Center Hospital East (*n* = 52, links = 62, total link strength = 363, Cluster 1), UT MD Anderson Cancer Center (*n* = 79, links = 56, total link strength = 144, Cluster 2), and Istituto di Ricovero e Cura a Carattere Scientifico (IRCCS) (*n* = 36, links = 39, total link strength = 127, Cluster 3). The largest cluster was Cluster 1, with 46 nodes representing different institutions (**Figure 4A**).

Among the 7,683 authors, 307 with more than five documents were included in the cooperation network analysis. In the author cooperation network map and overlay visualization, 26 clusters were formed (**Figure 4B**). The most productive authors in the top three clusters were Yoshino Takayuki (*n* = 34, links = 62, total link strength = 282, Cluster 1), Kopetz Scott (*n* = 46, links = 46, total link strength = 190, Cluster 2), and Aleshin Alexey (*n* = 17, links = 41, total link strength = 144, Cluster 3). The largest cluster was Cluster 1, consisting of 37 authors (**Figure 4B**).

#### 3.3.2 Co-occurrence network analysis

A total of 1,536 keywords were extracted from the 1,310 documents. The top 10 most frequent keywords, as shown in **Table 3A**, were as follows: “colorectal cancer” (freq = 558), “circulating tumor DNA” (freq = 489), “liquid biopsy” (freq = 350), “acquired resistance” (freq = 203), “cell-free DNA” (freq = 196), “plasma” (freq = 149), “mutations” (freq = 131), “metastatic colorectal cancer” (freq = 120), “therapy” (freq = 112), and “lung cancer” (freq = 111).

**Table 3A:** Most relevant words.

Freq	Degree	Centrality	Sigma	Label	Cluster ID
558	63	0.07	1	Colorectal cancer	0
489	62	0.06	1	Circulating tumor DNA	0
350	37	0.01	1	Liquid biopsy	0
203	64	0.04	1.43	Acquired resistance	2
196	60	0.05	1	Cell-free DNA	0
149	56	0.05	1	Plasma	3
131	52	0.04	1	Mutations	2
120	53	0.04	1	Metastatic colorectal cancer	0
112	53	0.04	1	Therapy	2
111	74	0.09	1	Lung cancer	0
106	42	0.03	1	Colon cancer	1
99	38	0.02	1	Survival	1
86	39	0.02	1	Evolution	2
80	69	0.07	1.32	Breast cancer	0
78	32	0.01	1.04	Heterogeneity	2
77	63	0.07	1	Circulating tumor cells	6
73	57	0.04	1	Blood	3
71	58	0.05	1	Cetuximab	2
69	33	0.02	1.08	Kras mutations	0
69	33	0.01	1	Ras mutations	2

Keywords related to detection technologies accounted for 4.60% (358/7,791) of the total frequency, with PCR-related keywords accounting for 43.64% (103/236) and next-generation sequencing (NGS) for 2.54% (6/236). The frequency ratio of keywords related to target genes was 11.08% (863/7,791), of which “BRAF” accounted for 10.00% (86/863), “EGFR” 12.05% (104/863), “KRAS” 23.18% (200/863), “Septin9” 1.85% (16/863), and “methylation” 14.14% (59/863) (**Table 3B**).

**Table 3B:** The frequency ratio of keywords.

Category	Keyword	Freq	Total	Ratio
Detection technology	PCR	103	236	0.436441
	NGS	6	236	0.025424
Target genes	BRAF	86	863	0.099652
	EGFR	104	863	0.12051
	KRAS	200	863	0.23175
	Septin 9	16	863	0.01854
	Methylation	122	863	0.141367

**Figure 5:**
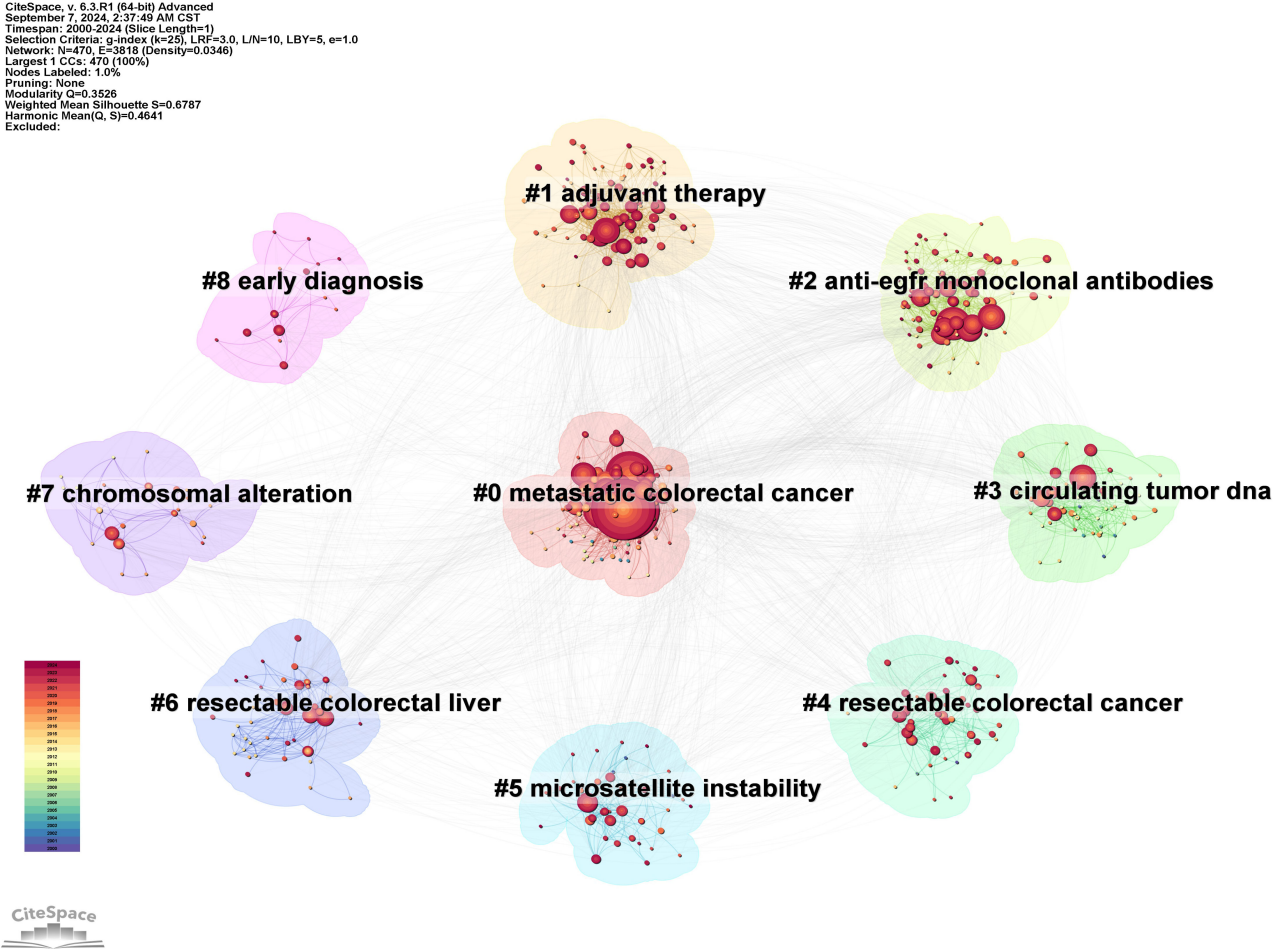
Keyword clustering analysis.

Nine main clusters were identified in the keyword network map generated by CiteSpace using the LLR algorithm for clustering analysis. These clusters included “metastatic colorectal cancer” (*n* = 100, Cluster 0), “adjuvant therapy” (*n* = 79, Cluster 1), “anti-EFGR monoclonal antibodies” (*n* = 76, Cluster 2), “circulating tumor DNA” (*n* = 50, Cluster 3), “resectable colorectal cancer” (*n* = 46, Cluster 4), “microsatellite instability” (*n* = 40, Cluster 5), “resectable colorectal liver metastases” (*n* = 40, Cluster 6), “chromosomal alteration” (*n* = 24, Cluster 7), and “early diagnosis” (*n* = 15, Cluster 8). Clusters 5 and 7 represented basic research on ctDNA associated with CRC, while Clusters 1, 2, 4, and 8 focused on clinical applications. Clusters 0 and 6 emphasized advanced CRC. The modularity index (*Q*) of the clustering was 0.3526 (>0.3), indicating that the cluster structure was significant, and the Silhouette (*S*) score was 0.6787 (>0.7), suggesting that the clusters were efficient and convincing (**Figure 5**). The overlap between different clustering blocks indicated relatively close internal connections. The largest cluster was Cluster 0, where “lung cancer” (degree = 74), “breast cancer” (degree = 69), and “cell lung cancer” (degree = 67) were the top three hub nodes. Similarly, in Cluster 1, the top three hub nodes were “1st line treatment” (degree = 61), “chemotherapy” (degree = 52), and “open-label” (degree = 47). In Cluster 2, the top three hub nodes were “acquired resistance” (degree = 64), “cetuximab” (degree = 58), and “therapy” (degree = 53) (**Supplementary Table 2**).

#### 3.3.3 Co-citation network analysis

The top 10 most cited documents globally are listed in **Table 4**. The most cited document, with 3,177 citations, was published in *Sci Transl Med* by Bettegowda C et al. in 2014. This study compared the quantities of ctDNA and circulating tumor cells (CTCs) in the same patients using an unbiased approach, concluding that ctDNA is a broadly applicable, sensitive, and specific biomarker that can be utilized for various clinical and research purposes in patients with multiple types of cancer.

**Table 4:** Top 10 most cited documents worldwide.

Rank	Paper	DOI	Total citations	TC per year	Normalized TC
1	Bettegowda C, 2014, *Sci Transl Med* (7)	10.1126/scitranslmed.3007094	3,177	288.81	8.79
2	Van Cutsem E, 2016, *Ann Oncol* (8)	10.1093/annonc/mdw235	2,307	256.33	18.07
3	Diehl F, 2008, *Nat Med* (9)	10.1038/nm.1789	1,921	113.00	1.00
4	Wan JCM, 2017, *Nat Rev Cancer* (10)	10.1038/nrc.2017.7	1,512	189.00	16.02
5	Crowley E, 2013, *Nat Rev Clin Oncol* (11)	10.1038/nrclinonc.2013.110	1,267	105.58	2.80
6	Siravegna G, 2017, *Nat Rev Clin Oncol* (12)	10.1038/nrclinonc.2017.14	1,186	148.25	12.57
7	Alix-Panabières C, 2016, *Cancer Discov* (13)	10.1158/2159-8290.CD-15-1483	954	106.00	7.47
8	Tie J, 2016, *Sci Transl Med* (14)	10.1126/scitranslmed.aaf6219	919	102.11	7.20
9	Siravegna G, 2015, *Nat Med* (15)	10.1038/nm.3870	664	66.40	6.91
10	Dienstmann R, 2017, *Nat Rev Cancer* (16)	10.1038/nrc.2016.126	620	77.50	6.57

**Figure 6:**
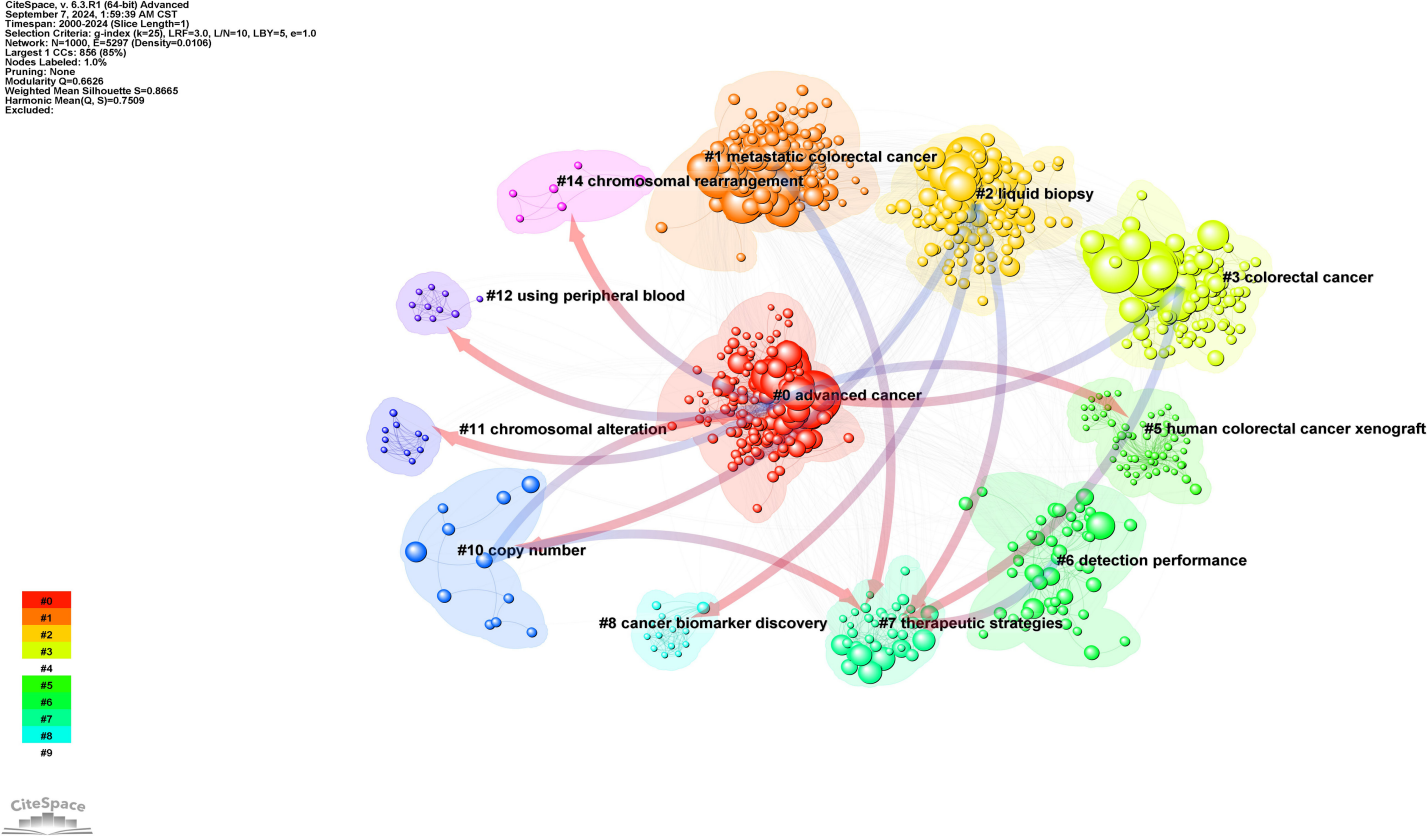
Reference co-citation cluster dependency.

**Figure 7:**
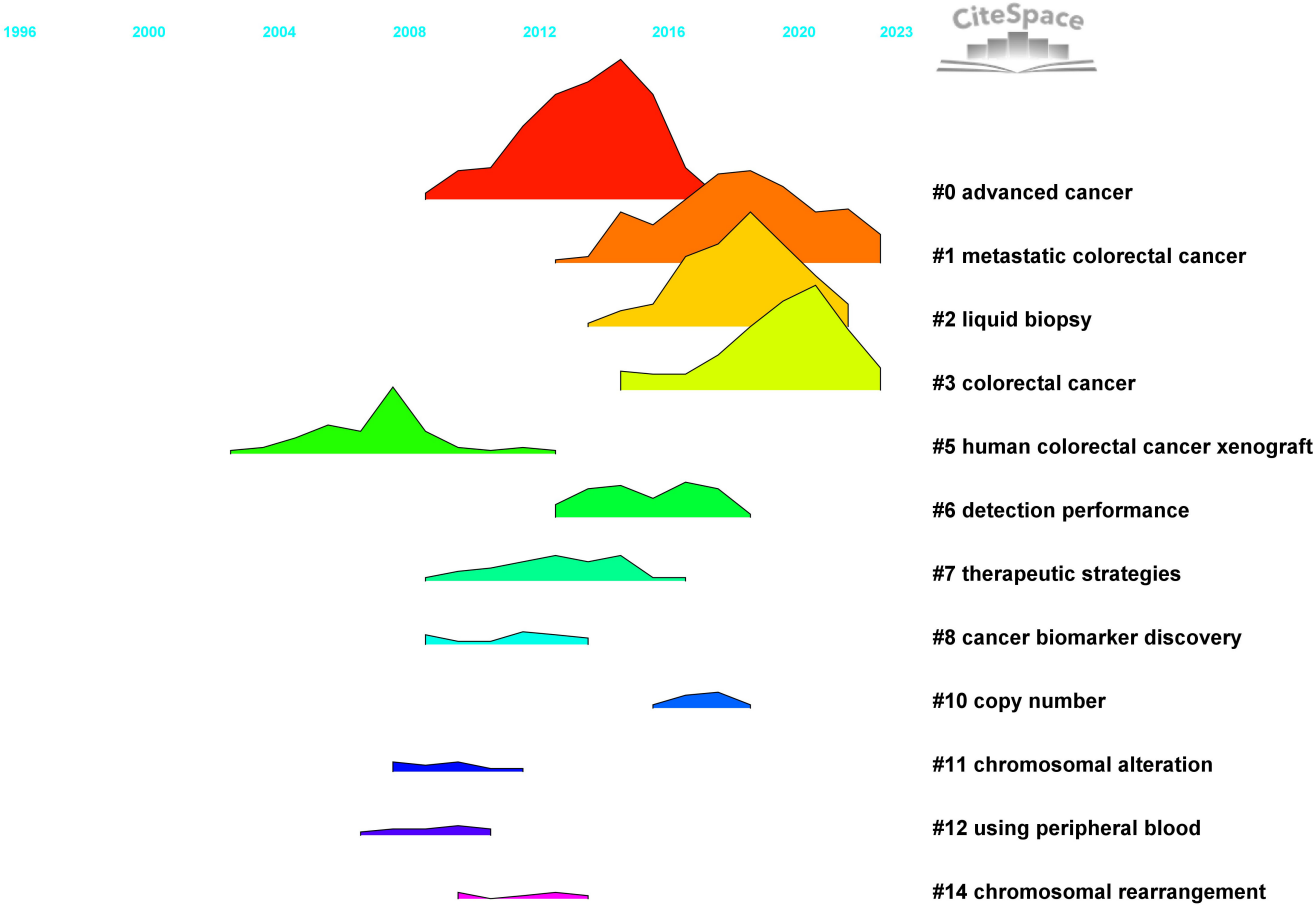
Landscape view of cited references.

A total of 29,970 references were extracted for co-citation network analysis using CiteSpace. The modularity index was 0.6626, and the Silhouette score was 0.8665, indicating that the cluster structure was significant and reliable (**Figure 6**). The overall structure in **Figure 7** illustrates how different integral clusters have evolved. Cluster 5, “human colorectal cancer xenograft,” started at the earliest and was the pioneering cluster. Clusters 0 (“advanced cancer”), 1 (“metastatic colorectal cancer”) 2 (“liquid biopsy”), and 3 (“colorectal cancer”), with higher peaks, have remained more active. Clusters 0 (“advanced cancer”), 1 (“metastatic colorectal cancer”), 2 (“liquid biopsy”), 3 (“metastatic colorectal cancer”), and 5 (“human colorectal cancer xenograft”) demonstrated longer time spans, indicating their persistence. Clusters 1 (“metastatic colorectal cancer”) and 3 (“colorectal cancer”), which extend into the most recent years, are still active today.

#### 3.3.4 In-depth analysis

**Figure 6** illustrates the dependency between the co-citation clusters and the main paths of direct citations. Cluster 0 cited Clusters 5, 10, 11, 12, and 14, while it was cited by Clusters 3 and 10. Cluster 2 cited Clusters 7, 8, and 10. Cluster 7 was cited by Clusters 1, 2, 3, 6, and 10. The main citation paths were from journals in molecular biology and genetics; to journals focused on health, nursing, and medicine; to journals related to medicine and clinical research. Additional paths were observed in molecular biology and genetics journals, as well as in those specializing in molecular biology and immunology.

**Figure 8:**
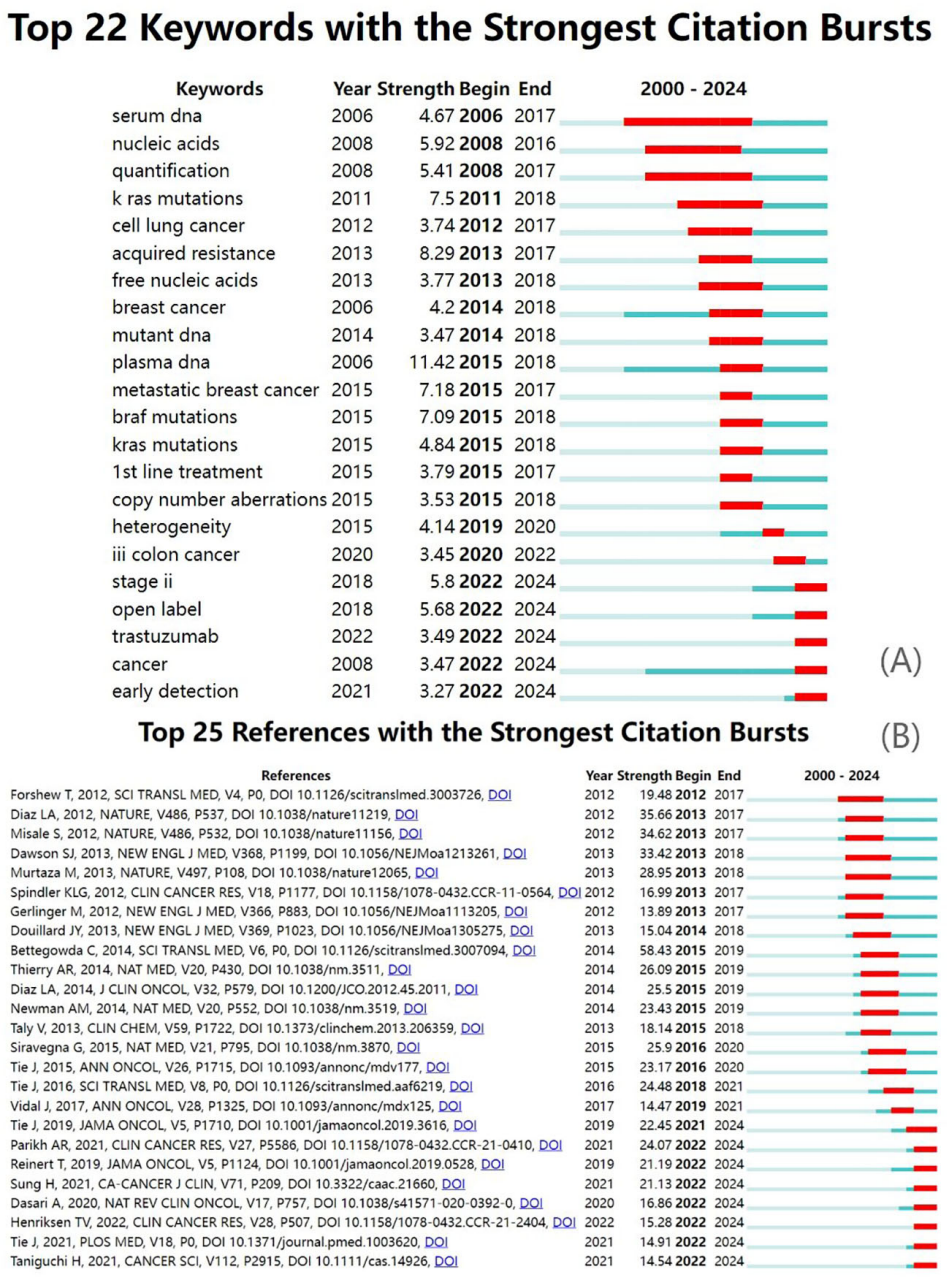
Burst analysis. **(A)** Keyword citation bursts; **(B)** reference citation bursts.

**Figure 9:**
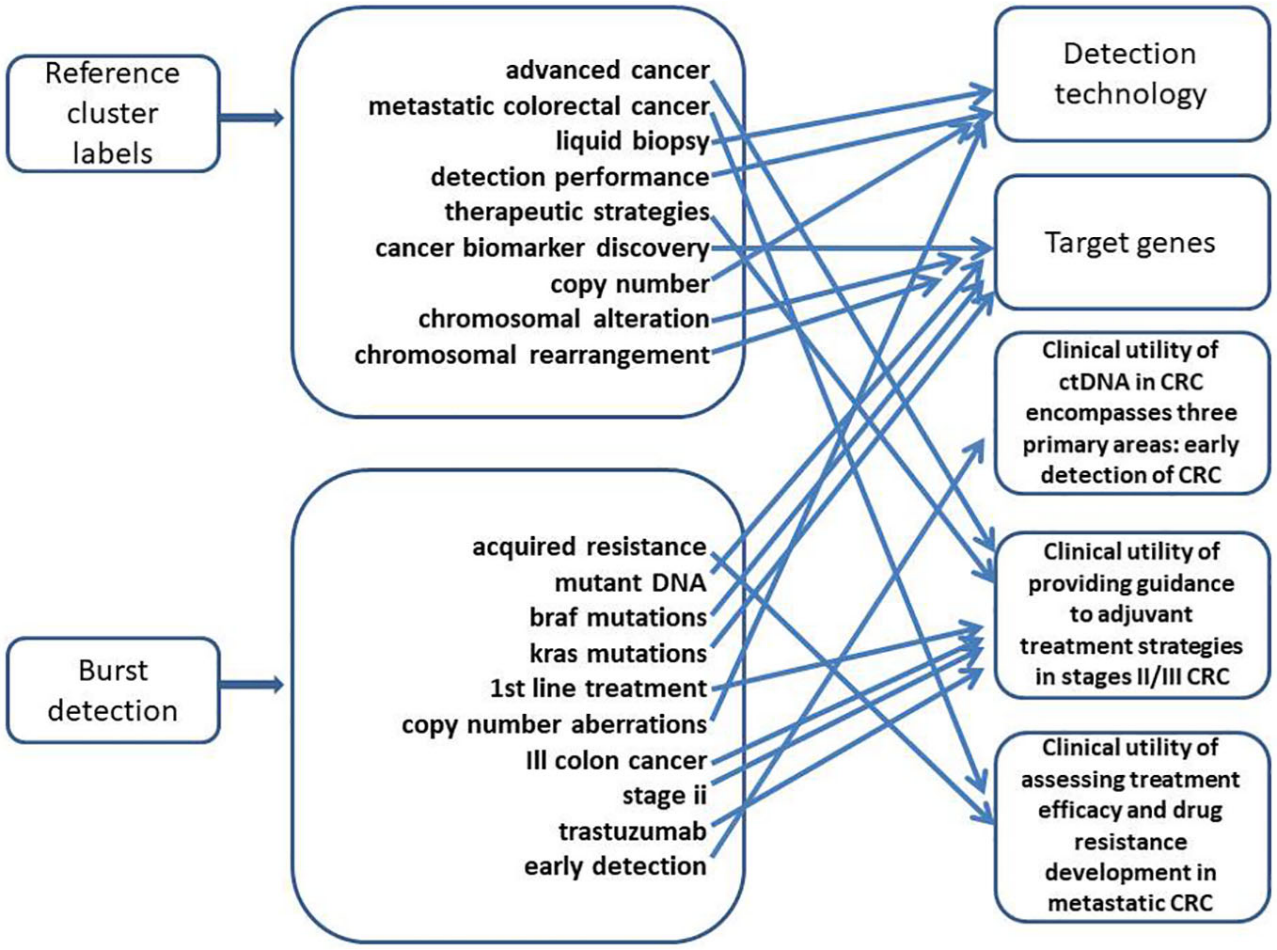
Research hotspots.

The top 22 keywords and top 25 references with the strongest citation bursts are shown in **Figures 8A, B** (**Supplementary Table 3**), highlighting the periods during which these keywords and references experienced a surge in citations. Keywords such as “early detection,” “stage II/III colorectal cancer,” “metastatic colorectal cancer,” and “treatment” demonstrated high sigma indices, indicating that clinical applications have been a prominent and hot research direction in recent years (**Figure 9**).

## 4 Discussion

The emerging economic and social burden posed by CRC presents an urgent challenge that demands prompt action. Research activity in this field correlates with CRC incidence and mortality rates. The top five most productive countries in this sense were found to be in Europe and Eastern Asia, where the disease incidence is high. Notably, not all of the most productive countries and journals were also the most cited, indicating that the quantity of publications does not always correspond to higher citation counts. The number of single-authored documents, the average number of authors per document, and the percentage of international co-authorships highlight this field’s complexity, its interdisciplinary nature, and the diversity of resources and skills required. This has led to a high degree of collaboration in CRC research. This trend is consistent with the increased formation of collaborative networks between different countries, institutions, and authors over the past two decades. Countries, institutions, and authors with more publications and citations were more likely to engage in cooperative efforts.

Keyword co-occurrence analysis is a valuable tool for examining the key topics within a research field. In the case of CRC, detection technology, genetic variation, and clinical applications emerged as the primary themes. Excluding search terms, “liquid biopsy” was the most frequently occurring keyword. As an evolving research area, liquid biopsy has already achieved significant milestones. Before the 1990s, liquid biopsy technology began to be explored. In the 1990s, deeper insights were gained into how cancer cells enter the bloodstream and their mechanisms for dissemination and survival. Between 2000 and 2010, liquid biopsy technology started to make its way into clinical practice. From 2010 to the present, liquid biopsies have played a significant role in various areas, including the early detection of CRC, guiding adjuvant therapeutic strategies for stage II/III CRC, and assessing treatment efficacy and the development of drug resistance in metastatic CRC.

By analyzing the keywords related to detection technology, it is evident that digital PCR (dPCR) and NGS are the most frequently used methods for ctDNA detection today. dPCR enables targeted gene amplification and quantification without a calibration curve based on a known number of samples. It offers advantages such as high sensitivity, speed, and cost-effectiveness; however, its limitations include a finite number of detectable targets. On the other hand, NGS is a powerful technology for DNA sequencing and genetic data acquisition, allowing for the simultaneous analysis of numerous short DNA sequences, which are then mapped or remapped to reference genomes. However, the sensitivity of NGS-based techniques is generally lower than that of PCR-based methods.

Moreover, the sensitivity of NGS is inversely related to the number of analysis sites (17). Given that ctDNA detection accuracy is influenced by factors such as tumor cell turnover, tumor size, tumor staging, and tumor mutation burden, the development of new detection technologies and equipment remains a crucial area of research. Recent studies have shown that AccuScan, a highly efficient cell-free DNA whole-genome sequencing technology, can detect ctDNA at concentrations as low as one part per million (18). Additionally, recent research has introduced a bimodal biosensor that employs a dual CRISPR-Cas12a system to quantify targets via fluorescence and electrochemical signals, explicitly targeting the epidermal growth factor receptor (EGFR) mutation L858R in specific non-small cell lung cancer (NSCLC) patients. This biosensor offers a dynamic detection range from 10 fM to 1 μM, with a detection limit of 372 aM. It demonstrates outstanding specificity, repeatability, stability, and recovery rate.

However, further extensive research is warranted to validate the advantages of these technologies in ctDNA detection in colorectal tumors (19). Consequently, we believe that in the future, the development of new technologies and equipment will focus not only on factors such as cost, yield, and specificity but also on the apoptosis, necrosis, and active release of cells at different stages of the CRC process. Additionally, the release and clearance mechanisms of ctDNA and overcoming technical limitations and biological noise through the integrated application of nanomaterial-based detection, machine learning techniques, artificial intelligence, and multi-omics approaches will be critical areas of future research (20).

Detection of ctDNA ranges from the analysis of individual mutations to comprehensive genome-wide studies. Research has shown that approximately 80 genes in each CRC cell carry mutations that are not found in normal cells. In line with our analysis of keywords related to target genes, the 14 most common genes, with more than 240 hotspots—primarily single-nucleotide variants (SNVs) and short insertions/deletions (Indels)—are ***AKT1**, **BRAF**, **CTNNB1**, **EGFR**, **ERBB2**, **FBXW7**, **GNAS**, **KRAS**, **MAP2K1**, **NRAS**, **PIK3CA**, **SMAD4**, **TP53***, and ***APC*** (21, 22). Molecular subtypes of CRC, identified through large-scale data analysis, have also been established. Four subtypes have been defined: CMS1 (14%, microsatellite instability with immune solid activation), CMS2 (37%, chromosomal instability with active WNT and MYC signaling), CMS3 (13%, metabolic dysregulation), and CMS4 (23%, characterized by significant TGF-β activation, stromal invasion, and angiogenesis). However, translating this classification into clinical practice is hindered by the requirement for specialized knowledge in gene expression profiling and associated costs and time (22).

Hub nodes in co-occurrence networks typically link different parts of a network and act as bridges between various research topics or clusters, playing a pivotal role in knowledge dissemination and information flow. Among the nine hub nodes identified in the top three clusters, the presence of lung cancer and breast cancer caught our attention. Traditionally, cancer research has been compartmentalized, focusing on a single type of cancer. However, owing to the shared mechanisms of cancer development, genetic variation, and treatment response, comprehensive studies of multiple cancer types—called Pan-cancer research—may help uncover broader biological patterns. This approach could lead to more effective methods for preventing, diagnosing, and treating CRC and other cancers.

Citation clustering analysis and the cross-citation of scientific literature reveal the objective patterns of scientific development, as well as the intersection and integration between disciplines. One model of knowledge development observed the merging of topics such as molecular biology and genetics with health, nursing, and medicine, culminating in the subject areas of medicine, medical research, and clinical practice. Another model demonstrated the independent progression of molecular biology and genetics toward the fields of molecular biology and immunology. Sudden bursts of keywords and references, such as “early detection,” “cancer,” “trastuzumab,” “open-label,” and “stage II,” highlight emerging research topics. Activity in these fields supports the translation of fundamental research discoveries into clinical applications, facilitating the advancement of medical practice.

Currently, the clinical utility of ctDNA in CRC encompasses three primary areas: early detection of CRC, providing guidance for adjuvant treatment strategies in stages II/III CRC, and assessing treatment efficacy and drug resistance development in metastatic CRC. In an average-risk screening population, a ctDNA-based blood test demonstrated a sensitivity of 83% for CRC detection, a specificity of 90% for advanced tumors, and a sensitivity of 13% for advanced precancerous lesions. The test’s specificity was negatively correlated with age, potentially due to age-specific characteristics of ctDNA methylation. In contrast, the sensitivity was positively associated with age, cancer staging, and the size and severity of advanced precancerous lesions (23).

Detecting abnormal DNA methylation, which occurs early in CRC development, is increasingly being implemented in clinical practice to diagnose early-stage CRC. Studies have shown that integrating methylation markers into ctDNA, such as ***SFRP1**, **SFRP2**, **SDC2***, and ***PRIMA1***, significantly improves the diagnostic accuracy for CRC, with some cases exhibiting sensitivities exceeding 90% (24). However, the American Society of Clinical Oncology (ASCO) and the College of American Pathologists (CAP) concluded that the current evidence supporting the clinical efficacy of ctDNA testing for CRC screening remains insufficient (25). Although studies have shown that the DNA fragment length distribution differs between healthy individuals and CRC patients, technological advancements still need to enhance sensitivity and reduce false-positive rates (26).

Minimal residual disease (MRD) refers to the minute quantity of malignant cells that remain after cancer therapy and can only be detected through highly advanced diagnostic methods. These residual cancer cells are so few that they evade detection by standard diagnostic techniques. However, ctDNA has a relatively short half-life, allowing for precise and real-time monitoring of disease progression. Combining epigenetic data with traditional genetic profiling can significantly enhance MRD detection accuracy, although a standardized approach has yet to be established (27). Some research suggests that assessing MRD using ctDNA outperforms all current risk stratification methods based on clinical pathology (28, 29).

In clinical practice, MRD evaluation is typically conducted 4 weeks or more after curative surgery and 2 weeks or more after the completion of systemic therapy. For longitudinal monitoring, ctDNA is usually assessed every 8 to 12 weeks. Numerous studies have confirmed that patients with MRD-positive and detectable ctDNA almost invariably experience disease recurrence if no additional treatment is administered (30). A collection of ctDNA profiling studies revealed that disease recurrence was detected, on average, 8.7 months earlier than with conventional radiological imaging (31).

In terms of assessing treatment efficacy, research indicates that ctDNA surpasses high PD-L1 expression, MSI-H, and TMB-H in dynamically monitoring the treatment response to immunotherapy, even in CEA-negative colorectal tumors (32). It is important to note that while elevated ctDNA levels are linked to poor prognosis in patients with metastatic cancer, ctDNA may not be as reliable in patients at high risk of peritoneal metastasis, as the location of metastatic disease may influence ctDNA levels (33). The use of ctDNA to identify alternative molecular mechanisms underlying resistance to targeted therapies primarily focuses on the MAPK signaling pathway, which includes ***KRAS**, **NRAS**, **BRAF**, **MAP2K1**, **MAP2K2**, **EGFR-ECD***, and amplifications of ***MET*** and ***HER2***. One of the main factors contributing to resistance in CRC is tumor heterogeneity. For example, the ***KRAS*** mutation, which leads to developed tolerance to EGFR inhibitors, has been recognized as a key driver of acquired resistance to cetuximab.

The National Comprehensive Cancer Network (NCCN) and the European Society of Medical Oncology recommend that CRC patients with ***BRAF*** oncogene mutations should not be treated with cetuximab or panitumumab (34). Research suggests that, even before the initiation of panitumumab therapy, resistance mutations in ***KRAS*** and other genes may already exist within clonal subpopulations of the tumor, leading to continuous mutation generation. The parallel evolution of distinct cellular clusters with different resistance mechanisms within the same metastatic lesion may explain the lack of response to anti-EGFR therapy, even in patients with ***KRAS*** wild-type tumors (35, 36). Current evidence does not suggest that alterations in ***PIK3CA*** and ***PTEN***, which are frequently observed alongside ***KRAS*** or ***BRAF*** mutations, can serve as reliable markers for resistance to EGFR antibody treatment. Because of the molecular complexity, variability, and heterogeneity of CRC, ctDNA provides a minimally invasive and potentially comprehensive method for monitoring dynamic changes during treatment. This is especially valuable for assessing ***RAS*** mutation status before and during anti-EGFR-directed therapies (37).

Although ctDNA has made significant strides in the early diagnosis, disease management, and monitoring of CRC, its clinical application still faces certain limitations. First, further comprehensive research and advancements in gene typing and genomic sequencing methods are essential to enhance detection sensitivity, reduce the incidence of false positives, and establish standardized procedures and universal criteria to optimize the clinical utility of ctDNA (38–40). Second, the use of ctDNA clearance rate measurements has not been thoroughly evaluated due to the lack of comprehensive studies. Additional research is needed to determine the optimal blood collection timeframe to accurately assess ctDNA levels (41). Concurrently, a substantial body of research is required to validate the feasibility of downgrading treatment strategies for patients with negative ctDNA results and to assess the efficacy of alternative adjuvant systemic therapies when MRD is detected following the completion of adjuvant chemotherapy (42). Finally, while some researchers have developed ctDNA response assessment criteria for solid tumors based on observed clinical responses and disease progression in various cancers—and have aligned these criteria with RECIST assessments—a consensus on standardized testing protocols and unified outcome evaluation criteria remains necessary (43). There were several limitations in our study. First, selection bias may arise from using a single database and language. However, the number of documents included was sufficient to represent the current state of ctDNA research related to CRC. Second, despite the abundance of data, variations in author names or inconsistencies in keyword expressions may have introduced bias. Third, our analysis focused solely on cooperation networks, co-occurrence networks, and co-citation networks, resulting in insufficient information mining. For example, we did not analyze the foundational aspects of the research, which could have provided deeper insights.

## 5 Conclusion

Over the past two decades, research on ctDNA has significantly influenced the diagnostic and therapeutic strategies for CRC. Since 2012, there has been a notable increase in the volume of literature focusing on ctDNA in the context of CRC. The USA, China, Italy, Japan, Spain, and Australia have substantially contributed to advancing this field. The collaborative networks mainly comprise highly productive authors, renowned institutions, and leading countries. Interest in ctDNA for CRC continues to grow, accompanied by improvements in the quality of research in this area. We firmly believe that advancements in detection technologies, the development of standardized protocols, the investigation of ctDNA tumor dynamics in CRC, and the execution of large-scale clinical trials for ctDNA-guided precision therapy in CRC are likely to become primary future research directions.

## Data Availability

The raw data supporting the conclusions of this article will be made available by the authors, without undue reservation.
