# Supplementary material for: Bibliometric analysis of global research trends and prospects on circulating tumor DNA in colorectal cancer
Source: Front Oncol. 2024 Nov 15;14:1428942. doi: 10.3389/fonc.2024.1428942 (PMC11604578; doi:10.3389/fonc.2024.1428942)
Supplement: Supplementary file 1 [file Table1.docx]

Supplementary Table 1：Annual production

| **Year** | **Articles** |
| --- | --- |
| 2000 | 1 |
| 2001 | 2 |
| 2002 | 0 |
| 2003 | 1 |
| 2004 | 1 |
| 2005 | 0 |
| 2006 | 1 |
| 2007 | 0 |
| 2008 | 1 |
| 2009 | 0 |
| 2010 | 1 |
| 2011 | 3 |
| 2012 | 3 |
| 2013 | 5 |
| 2014 | 11 |
| 2015 | 32 |
| 2016 | 57 |
| 2017 | 78 |
| 2018 | 139 |
| 2019 | 152 |
| 2020 | 159 |
| 2021 | 192 |
| 2022 | 222 |
| 2023 | 216 |
| 2024 | 33 |

Supplementary Table 2 Hub nodes

| Freq | Burst | Degree | Centrality | Sigma | Label | ClusterID |
| --- | --- | --- | --- | --- | --- | --- |
| 111 | 0 | 74 | 0.09 | 1 | lung cancer | 0 |
| 80 | 4.2 | 69 | 0.07 | 1.32 | breast cancer | 0 |
| 36 | 3.74 | 67 | 0.07 | 1.3 | cell lung cancer | 0 |
| 49 | 3.79 | 61 | 0.05 | 1.2 | 1st line treatment | 1 |
| 67 | 0 | 52 | 0.05 | 1 | chemotherapy | 1 |
| 61 | 5.68 | 47 | 0.04 | 1.22 | open label | 1 |
| 203 | 8.29 | 64 | 0.04 | 1.43 | acquired resistance | 2 |
| 71 | 0 | 58 | 0.05 | 1 | cetuximab | 2 |
| 112 | 0 | 53 | 0.04 | 1 | therapy | 2 |

Supplementary Table 3 Important keywords

| Freq | Burst | BurstBegin | BurstEnd | Degree | Centrality* | Sigma** | Label | ClusterID |
| --- | --- | --- | --- | --- | --- | --- | --- | --- |
| 203 | 8.29 | 2013 | 2017 | 64 | 0.04 | 1.43 | acquired resistance | 2 |
| 31 | 7.18 | 2015 | 2017 | 49 | 0.04 | 1.33 | metastatic breast cancer | 0 |
| 80 | 4.2 | 2014 | 2018 | 69 | 0.07 | 1.32 | breast cancer | 0 |
| 68 | 11.42 | 2015 | 2018 | 45 | 0.02 | 1.32 | plasma dna | 0 |
| 36 | 3.74 | 2012 | 2017 | 67 | 0.07 | 1.3 | cell lung cancer | 0 |
| 61 | 5.68 | 2022 | 2024 | 47 | 0.04 | 1.22 | open label | 1 |
| 26 | 7.5 | 2011 | 2018 | 44 | 0.03 | 1.22 | k ras mutations | 0 |
| 49 | 3.79 | 2015 | 2017 | 61 | 0.05 | 1.2 | 1st line treatment | 1 |
| 12 | 3.77 | 2013 | 2018 | 36 | 0.03 | 1.12 | free nucleic acids | 0 |
| 17 | 5.92 | 2008 | 2016 | 41 | 0.02 | 1.11 | nucleic acids | 0 |
| 69 | 4.84 | 2015 | 2018 | 33 | 0.02 | 1.08 | kras mutations | 0 |
| 27 | 7.09 | 2015 | 2018 | 26 | 0.01 | 1.08 | braf mutations | 2 |
| 39 | 5.8 | 2022 | 2024 | 32 | 0.01 | 1.06 | stage ii | 1 |
| 33 | 5.41 | 2008 | 2017 | 31 | 0.01 | 1.06 | quantification | 7 |
| 23 | 3.47 | 2022 | 2024 | 31 | 0.02 | 1.06 | cancer | 5 |
| 78 | 4.14 | 2019 | 2020 | 32 | 0.01 | 1.04 | heterogeneity | 2 |
| 8 | 3.47 | 2014 | 2018 | 21 | 0.01 | 1.02 | mutant dna | 0 |
| 12 | 3.27 | 2022 | 2024 | 19 | 0.01 | 1.02 | early detection | 4 |
| 18 | 3.45 | 2020 | 2022 | 21 | 0 | 1.01 | iii colon cancer | 1 |
| 9 | 3.49 | 2022 | 2024 | 15 | 0 | 1.01 | trastuzumab | 5 |

Centrality*: Betweenness Centrality, refers to the frequency with which a node appears on the shortest paths between all pairs of nodes in a network. It can be used to identify key nodes in the network that may play a significant role in information transmission, resource allocation, or the spread of influence. Sigma**: a composite indicator that combines the node's betweenness centrality and burstiness. The calculation method is: Sigma = (Centrality + 1) ^burstness,^.
